# Characterization of a PCV2d-2 isolate by experimental infection of pigs

**DOI:** 10.1186/s12985-018-1098-0

**Published:** 2018-11-27

**Authors:** Vilmos Palya, Zalán G. Homonnay, Tamás Mató, István Kiss

**Affiliations:** Scientific Support and Investigation Unit, Ceva-Phylaxia, Szállás u. 5, Budapest, 1107 Hungary

**Keywords:** PCV2d-2, Viremia, Excretion, Virus neutralization

## Abstract

Porcine circovirus type 2 (PCV2), a highly prevalent, economically important swine pathogen is classified into different genotypes (PCV2a-f) based on phylogenetic analysis. Since the introduction of extensive vaccination programs, at least two major shifts have been observed in the prevalence of PCV2 genotypes. The first genotype shift from 2a towards 2b occurred around 2003, while in recent years, we are witnessing the second change in genotype prevalence from the predominant 2b towards 2d.

In this study, a PCV2d-2 isolate was characterized as a potential challenge virus for the evaluation of PCV2 vaccine efficacy. Ten-week-old pigs carrying low to moderate levels of maternally derived antibodies to PCV2 were infected with the isolate by the nasal route. Over the next 4 weeks post-infection, the pigs were monitored for the presence of viremia, fecal virus excretion, and humoral immune responses. At the end of the post-infection observation period, samples were taken from the mediastinal and mesenteric lymph nodes of the animals and tested for viral load. The gradual depletion of maternally derived antibodies in the sera of piglets was demonstrated by ELISA and virus neutralization tests. Following experimental infection by PCV2d-2, specific IgM antibodies were first detected at 14 days post challenge (dpch), while IgG class antibodies were first detected at 21 dpch. Both viremia and virus shedding could be detected at 7 dpch, in 36 and 50% of the pigs, respectively. The proportion of shedders reached 100% by 14 dpch and remained at this level, while viremia was demonstrated in 86, 100, and 100% of the pigs at 14, 21, and 28 dpch, respectively. Both the mediastinal and mesenteric lymph nodes contained high levels of virus (7.6 and 8.5 log_10_ copies/mg tissue, respectively).

## Main text

Porcine circovirus type 2 (PCV2) was first described in 1998 [1], and since then, it has become one of the most important pig pathogens, contributing to considerable economic losses by a syndrome called porcine circovirus disease (PCVD). The virus has demonstrated the highest evolution rate among similar DNA viruses, resulting in genetic shifts even over the rather short period of time since its recognition [2]. Today six genotypes of PCV2 are known, PCV2a-f [3]. PCV2d is the most widespread genotype [4], which is further subdivided into PCV2d-1 and 2 [5]. Pathogenicity of PCV2 is complex, and presumably involves the infection of pig fetuses, especially their thymus, where the virus establishes a latent infection. Changes of this fetal viral pool due to environmental factors such as stress, vaccination and last but not least, the genetics of the host, will determine the outcome of the infection [6].

In the field, PCVD is controlled by vaccination and intensifying the biosecurity measures on pig farms, and by the development of high hygiene systems of environmental control based on the recommendations of Madec et al. [7]. Current vaccines, comprising PCV2a strains or their proteins, have proven effective for protecting against clinical disease in pigs when challenged with PCV2a, PCV2b, or PCV2d, especially in controlled conditions [2]. Evaluating vaccines presumes access to appropriate challenge strain(s). Therefore, the aim of this study was to characterize a PCV2d-2 isolate, by using parameters such as viremia, virus shedding, and viral load in lymph nodes. Also, different assays to monitor humoral immune responses after challenge were assessed.

## Methods

### Source of virus isolate

In January 2016, organ samples of 10 week-old piglets were submitted for diagnostic investigation to our lab from a 1500 sow farrow-to-finish farm, having a clinical history of ill thrift and uneven condition. Necropsy was performed at the farm which revealed wasting, bronchopneumonia and chronic pleurisy. PCV2 was suspected as predisposing agent for subsequent secondary infections. To detect and isolate the virus, tissue samples of lungs and lymph nodes were collected.

For virus isolation, 10% *v*/*w* tissue homogenate of the mediastinal lymph nodes was prepared in PBS. After clarification by low speed centrifugation (1000 rpm for 10 min), 100 μL of the supernatant was inoculated onto 10^6^cell/T25 PK-IV-D56 cells. The inoculated cells were incubated at 37 °C with 5% CO_2_ and passaged at a 1 to 4 dilution rate four times, at 4–5 days intervals. After the last passage, the cell cultures were frozen at -20 °C. After three freeze-thaw cycles, the culture medium was collected and centrifuged at 3000 rpm for 10 min. The resulting supernatants were aliquoted. The identity of the isolate was confirmed during passages by comparing the nucleotide sequence of the passaged virus to the nucleotide sequence of the original virus detected in the lymph nodes.

### Diagnostic PCRs

The lymph node samples were tested for the presence of PCV2 [8], PCV3 [9], PRRSV (ADI132–100-ADIAVET PRRSV EU/NA REAL TIME PCR kit) and *M. hyopneumoniae* [10]. The purity of the PCV2 isolate after the passages was tested for the presence of Mycoplasmas, porcine parvoviruses, PCV-1 and PRRSV (EU1, EU2 and NA subtypes) by Vet-Med-Labor Ltd. (Budapest, Hungary).

The presence and level of virus in the tissue culture supernatant during the isolation process was monitored by qPCR [8].

### Challenge inoculum preparation

To prepare inoculum for the challenge experiment, the virus was passaged nine times as described above.

### Nucleotide sequencing, phylogenetic analysis

The whole genome of isolate D3276/5/16HU; (GenBank accession number: MG833033) was amplified using CBB1, CBB2, CBB3, and CSZ2 primers [11]. The nucleotide sequence was determined by Biomi Ltd. (Gödöllő, Hungary). Related PCV2 DNA sequences were obtained from NCBI GenBank using BLAST. DNA sequences were aligned and phylogenetic analyses were performed using MEGA 7.0 software [12].

### Pre-screening of the piglet source herd

The piglets (Seghers breed) originated from a farrow-to-finish farm in Szabolcs-Szatmár-Bereg county (Hungary). No PCV2 vaccination was used at the facility because it was considered a “low PCV2 pressure” farm. Furthermore, the farm was free of PRRSV, *M. hyopneumoniae*, and influenza A viruses according to regularly performed monitoring by PCR and serology. Before selecting the piglets for the study (at two weeks of age), sows and their litters were tested for viremia and fecal shedding of PCV2 (see details below).

### Piglets and housing

Twenty-two, 15–17 day old piglets of either gender, originating from sows found negative for PCV2 viremia and fecal shedding were transported to the trial facilities and kept on straw bedding in a pen. The piglets were fed according to their age and provided water ad libitum throughout the trial.

### Experimental challenge infection and sampling

Serological testing was performed at 3 weeks of age to check the level of residual MDA to PCV2, then right before challenge, and at weekly intervals for 4 weeks post-challenge. The pigs were challenged at ten weeks of age by administering 3 mL of challenge virus (10^3.1^TCID50/100 μL) into each nostril (6 mL/pig).

The applied challenge dose (1X10^3.1^TCID_50_/100 μL; 6 ml/pig) was in line with those published in the literature, i.e., 3 mL (1X10^3.55^TCID_50_/pig) and 3 mL (1X10^4.7^TCID_50_/pig), [13]; 2 mL of 10^4.5^TCID_50_/mL/pig [14].

The pigs were observed for 4 weeks for clinical signs and sampled weekly for fecal shedding, viremia, and isotype specific humoral immune responses.

At the end of the trial, the pigs were slaughtered at an abattoir. Swab and serum samples were collected. At the same time, mesenteric and mediastinal lymph nodes were collected for viral load measurements. Approximately 10 mg of the lymph nodes were processed for qPCR.

The identity of the challenge virus was checked by nucleotide sequence analysis of the detected PCV2 originating from separate pigs and comprising of pre-challenge serum samples, mediastinal and mesenteric lymph node samples collected at the slaughterhouse at the end of the trial.

### Serological investigations

For the screening of the sows providing the piglets for the study, SERELISA PCV2 Ab Mono Blocking kit (Zoetis) was used according to the manufacturer’s instructions. The immune response of the pigs to the challenge infection was measured by using the following assays:

1) SERELISA PCV2 Ab Mono Blocking kit; 2) BioChek Porcine Circovirus type 2 Antibody Test kit; 3) INGEZIM CIRCO IgG 11.PCV.K1; 4) INGEZIM Circovirus IgM/IgG ELISA kit (INGENASA), and 5) a modified virus neutralization assay (see below). The first three ELISAs and the VN assa were used on serum samples collected at 3, 10 (pre-challenge), and 14 weeks of age (4 weeks post challenge) while the INGEZIM IgM/IgG ELISA was used only to test the serum samples collected during the post-infection period in order to monitor the Ig isotype specific responses upon challenge.

In the VN assay the above-mentioned sera were checked for their ability to neutralize the PCV2b Rm strain (Ceva-Phylaxia Ltd.), which replicates more readily on cell culture than the PCV2d-2 isolate. Serial two-fold dilutions (in the range of 10–20,480X) were prepared from the sera and 500 μL of each serum sample was incubated with 500 MOI of PCV2/well on a 96-well plate (Falcon) for 1 h at 37 °C. Then, 10^5^ PI-IV-D56 cells (a PK-15 derived cell line) were added to each well and incubated for 5 days at 37 °C. The plates were frozen at -20 °C overnight before reading. The reading was performed by an antigen detecting sandwich ELISA. Briefly, the plates were coated with monoclonal antibody 36F1 (INGENASA, specific to VP2 of PCV2), incubated overnight at 4 °C, and then washed with PBS. Next, 50 μL of supernatant from each well of the neutralization test plates and 50 μL of PBTN (PBS with 0.05% Tween 20 and 0.02% sodium azide) was added and incubated for 1.5 h at 37 °C. Then the conjugate (INGENASA Anti PCV2 36F1 conjugated with 60 M biotin) was added and incubated for 1 h at 37 °C. Next, streptavidin peroxidase was added, followed by adding a mixture of tetramethyl-benzidine and hydrogen peroxide as substrate. The reaction was stopped by the addition of sulphuric acid and the absorbance was measured at 450 nm. Samples with an optical density (OD) > 2X the mean OD of a cell control were considered positive. The titer of the tested serum sample was determined by using the Spearman-Kärber method [15].

### Quantitative real-time PCR

Sera, rectal swabs, mediastinal and mesenteric lymph nodes of the experimentally infected pigs were tested by quantitative PCR (qPCR). Viral DNA was extracted by the QIAamp 96 DNA QIACube HT Kit with QIACube HT device (Qiagen) according to the manufacturer’s instructions. The qPCR assays were carried out according to Brunborg et al. [8] using QuantiNova Probe PCR Kit in a Rotor-Gene Q instrument (Qiagen). PCV2 virus suspension of known titer and a reference plasmid containing PCV2 sequences were used to quantify PCV2 virus load. For each reaction, the base-line and cycle threshold (Ct) number was determined automatically. A positive cut-off value of 36.7 Ct was determined. The PCV2 copy number of the samples was calculated using a 10-fold dilution series of a stock solution of a plasmid DNA containing PCV2 DNA sequences.

### Statistical analysis

The sensitivity and specificity of each ELISA kit used were calculated by comparing the results to the VN results, considered as reference of true positivity.

Regression analysis was performed between the VN and ELISA titers, the serological data and post-challenge viremia or virus excretion values (Ct), and the virus content of the mesenteric and mediastinal lymph nodes by using the STATGRAPHICS Centurion XVI (version 16.2.04) and Microsoft Excel software programs.

## Results

### Identification of the PCV2 isolate D3276/5/16HU

The diagnostic PCRs provided positive results for PCV2 and *M. hyopneumoniae* of the lymph node samples submitted from the clinical case. Upon serial passages on PKIV-D56 cells the 10th passage virus pool was positive for PCV2 and free from common swine viral pathogens and bacteria.

**Fig. 1:**
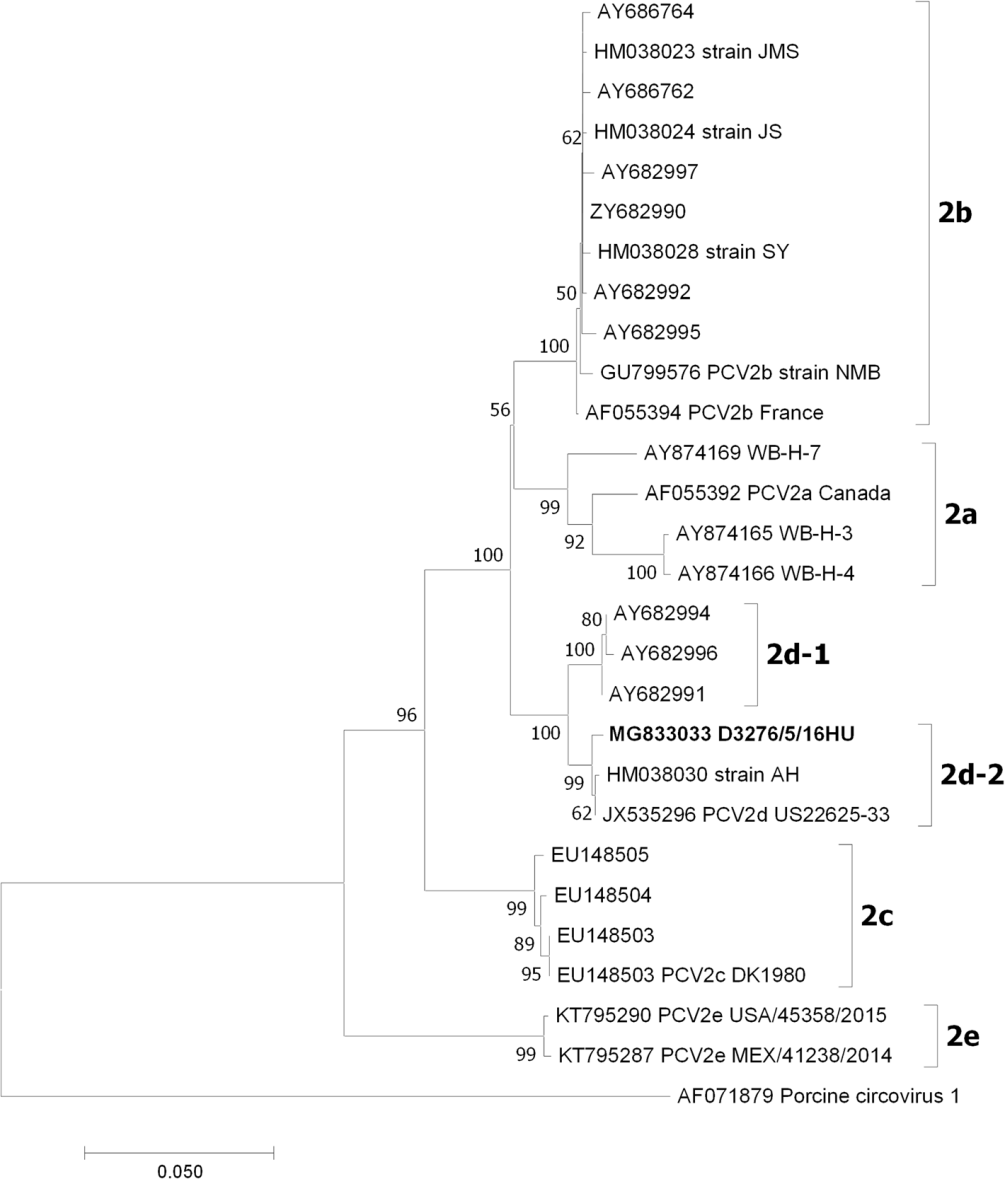
Phylogenetic tree of the ORF2 nucleotide sequences of PCV strains. The evolutionary history was inferred using the Neighbor-Joining method [22]. The percentage of replicate trees in which the associated taxa clustered together in the bootstrap test (500 replicates) are shown next to the branches [23]. The tree is drawn to scale, with branch lengths in the same units as those of the evolutionary distances used to infer the phylogenetic tree. The evolutionary distances were computed using the Maximum Composite Likelihood method [24] and are in the units of the number of base substitutions per site. The analysis involved 189 nucleotide sequences. Evolutionary analyses were conducted using MEGA7 [12]

The isolate was identified as PCV2d-2 [5] by phylogenetic analysis (Fig. 1). Its 1767 nt long DNA was coding for two open reading frames, ORF1 (314 aa, 942 nt) and ORF2 (234 aa, 702 nt). The ORF2 was coding for all the amino acids considered as PCV2d-specific [4], e.g. Phe8, Ile53, Lys59, Asn68, and Lys234.

Sequence analysis revealed that the nucleotide sequence of the D3276/5/16HU isolate had not changed throughout the passages and during the experimental infection of the pigs.

### Source farm screening

All 5 sows, from which the trial piglets were selected, proved negative by qPCR for viremia and fecal shedding and had calculated mean PCV2 antibody titer of 556 in the SERELISA test (cutoff 350).

### Post-challenge clinical observations

No clinical signs related to PCVD were observed among the pigs throughout the trial.

### Laboratory findings

#### Serology

**Fig. 2:**
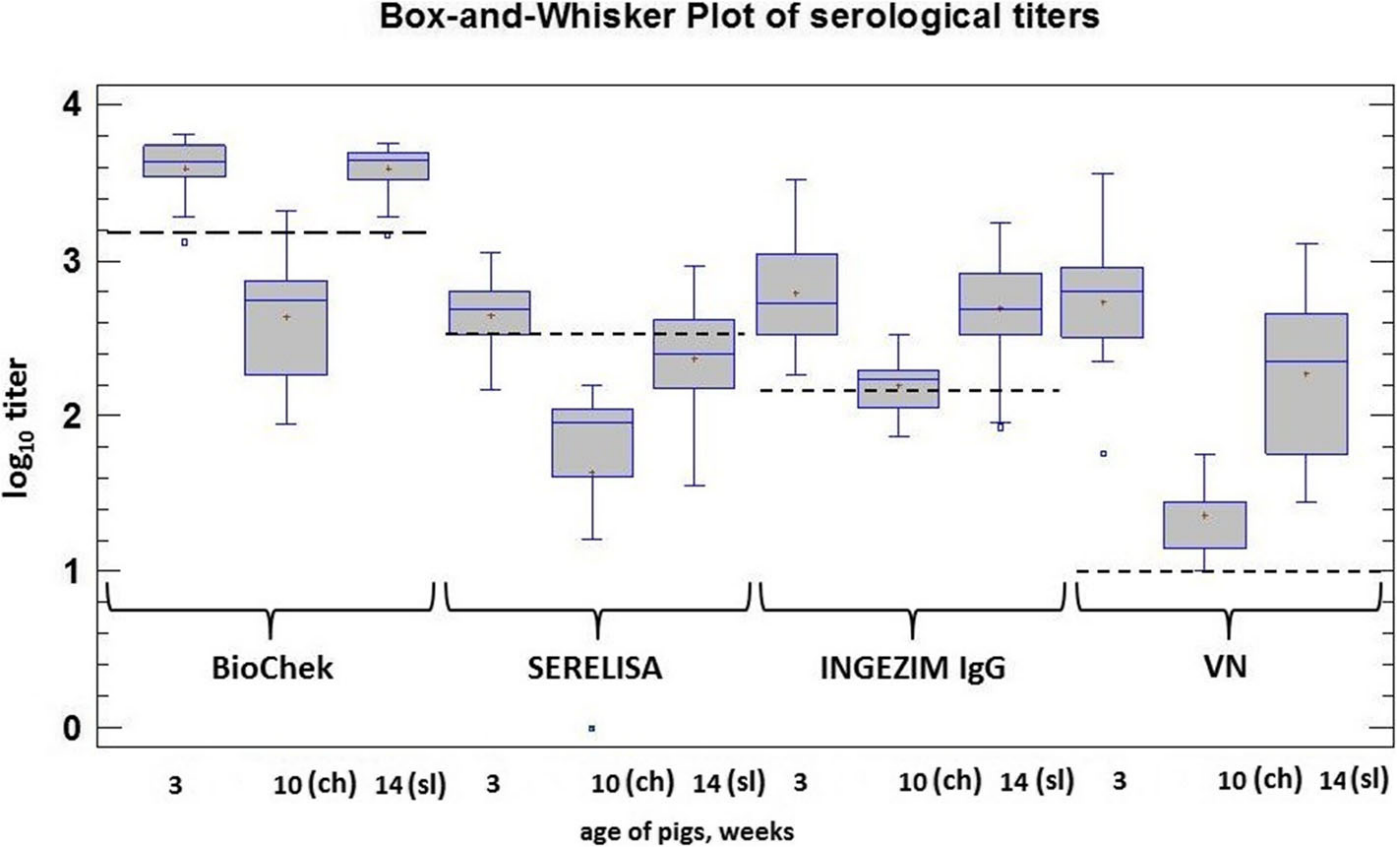
Box-and-Whisker Plot of serological titers of pigs over time using three commercial ELISA kits and a virus neutralization assay. A box represents the middle 50% of the data values. The continuous horizontal line within the box is the sample median. Plus indicates sample mean. Whiskers indicate the largest and smallest values. “Outside points” indicate values that are more than 1.5 times the interquartile range above or below the limits of the box. Discontinuous lines represent the positive threshold. ch = challenge; sl = slaughter

All serological assays indicated the presence of maternally-derived antibodies (MDA) in the piglets at 3 weeks of age, which by the time of challenge was nearly depleted. While most (BioChek) or all (SERELISA) serum samples were negative in the ELISAs, the presence of residual neutralizing MDA remained detectable by the VN assay at this time point (Fig. 2.).

The Durbin-Watson (STATGRAPHICS) analysis indicated a significant relationship between VN and calculated ELISA titers (*p* < 0.05). The Spearman’s rank correlation (MS Excel) test resulted in correlation coefficient values of 0.73, 0.59, and 0.72 for BioChek, SERELISA, and Ingenasa IgG kits and between VN (*p* < 0.05), respectively. The sensitivity was 92.19, 34.38, and 82.81%, the specificity 100, 100, and 50% for the BioChek, SERELISA, and Ingenasa IgG ELISA kit, respectively.

**Fig. 3:**
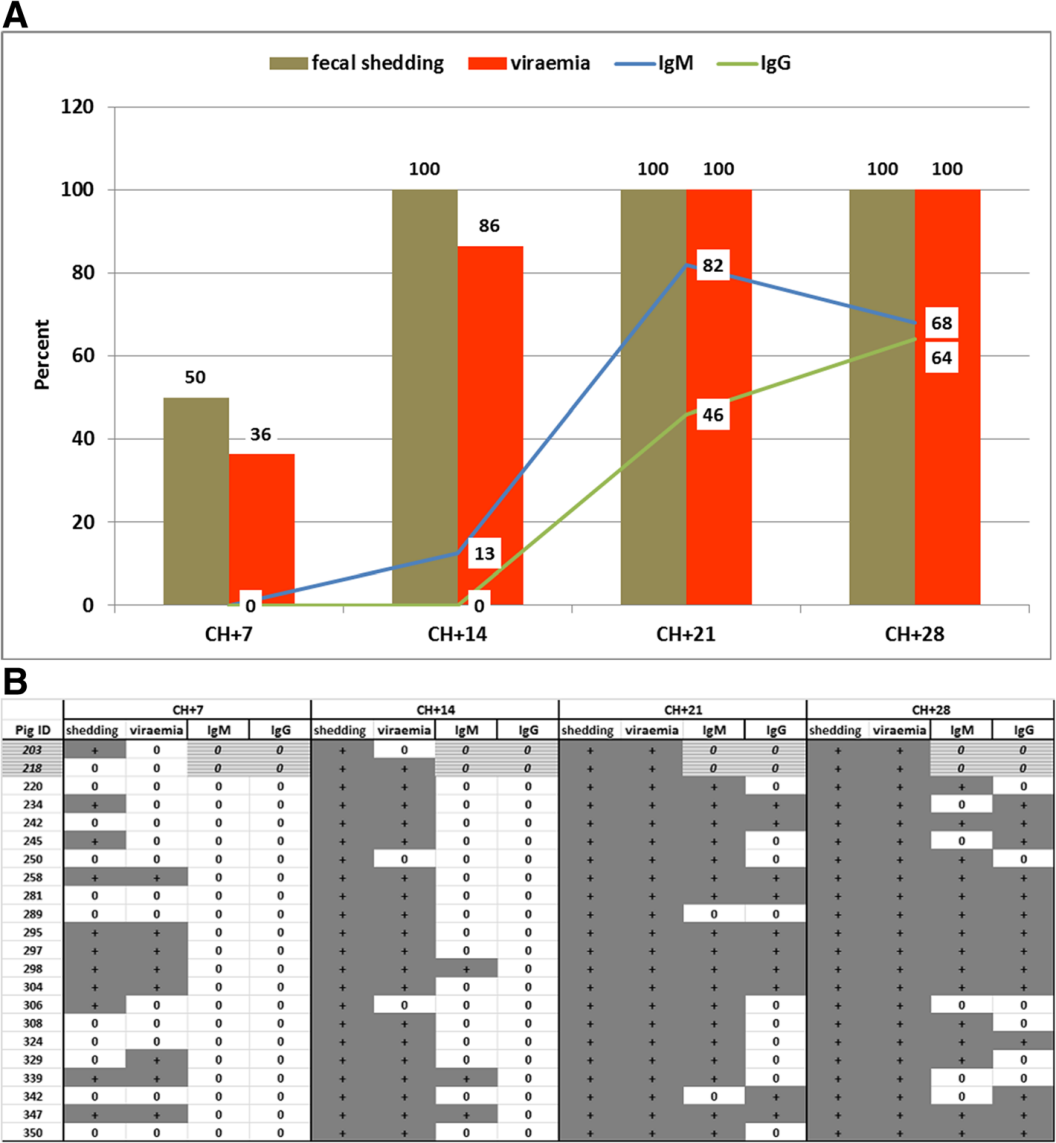
Summary of the kinetics of the experimental PCV2 infection demonstrating the percent of pigs positive for fecal shedding, viremia, PCV2 IgM and IgG antibodies. A, percent of positive pigs; B, individual data

The post-challenge follow up of the isotype specific humoral immune responses by using the INGEZIM ELISA indicated the development of IgM antibodies in 3 pigs (13%) by 14 dpch, which increased to 82% (18 pigs) by 21 dpch, then decreased to 68% (15 pigs) by 28 dpch. IgG response was not detected until 21 dpch, when 10 pigs (46%) were positive, which further increased to 14 (64%) by 28 dpch (Fig. 3a).

Two pigs remained negative for both INGEZIM IgM and IgG isotype specific ELISAs throughout the post-challenge observation period (Fig. 3b).

#### qPCR

**Fig. 4:**
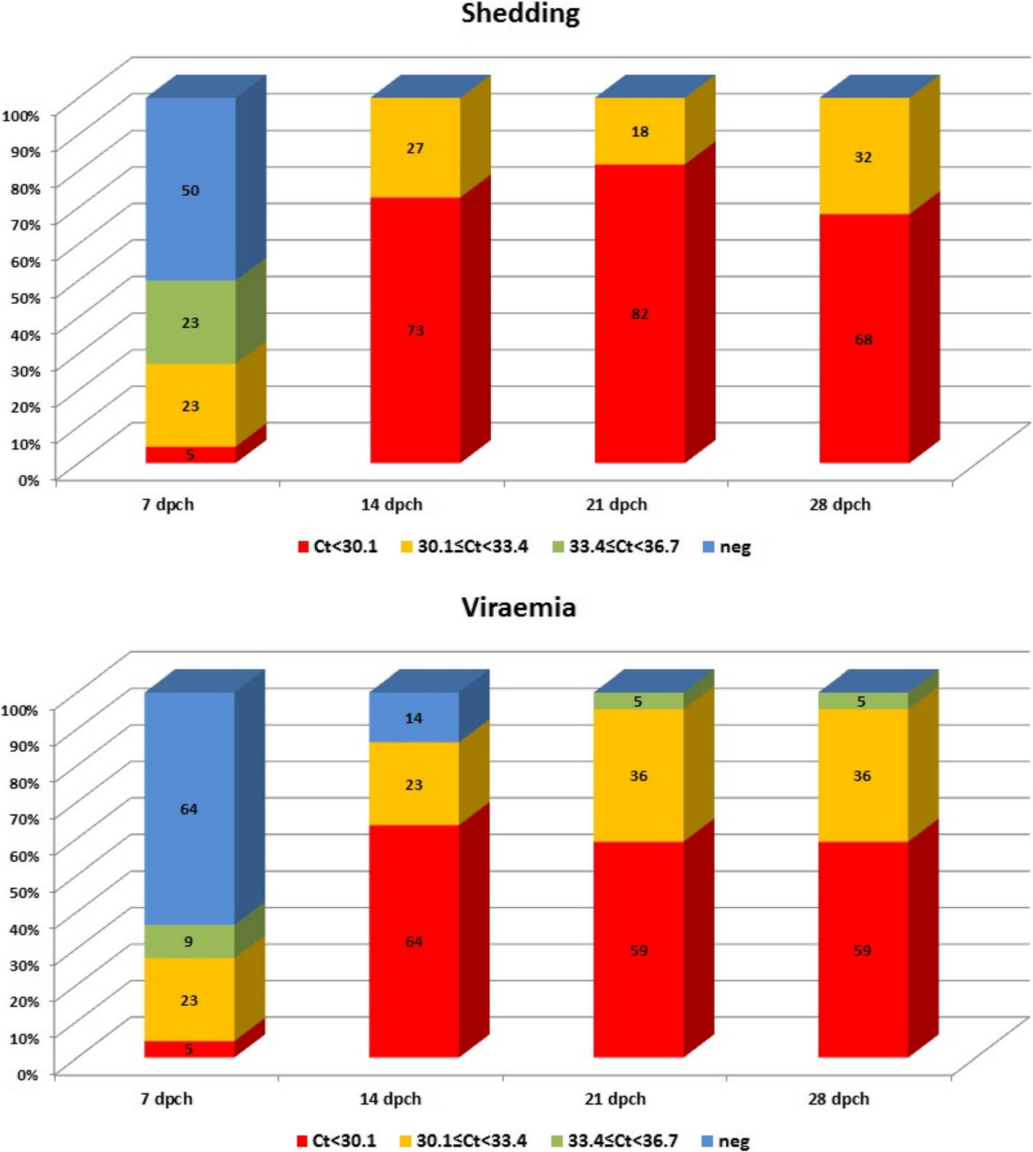
Post-challenge virus shedding and viremia results expressed as percent of pigs belonging to Ct categories reflecting one log difference of viral copy numbers

Viremia and fecal shedding were monitored in the experimentally infected animals for 28 dpch and quantified using a log_10_ standard curve generated from a PCV2 DNA plasmid construct (Fig. 4). The analysis of viremia and fecal shedding demonstrated that both the proportion of qPCR positive animals and the level of the virus increased in the serum and swab samples over time. Although 50% of the pigs shed the virus and 36% were viremic at 7 dpch, the percentage of strong qPCR positives (i.e. Ct < 30.1; ~ 36,700 target copy/microL) was rather low (5% for each type of sample). Over time, the proportion of samples with high viral loads (i.e. low Ct value in PCV2 specific qPCR) increased, and in case of fecal swabs there were no samples with a Ct value above 33.4 (~ 7300 target copy/microL) from 14 dpch onward.

The lymph nodes collected at the end of the study showed a high viral load as measured by qPCR (7.6 and 8.5 log_10_ target copy/mg tissue in the mediastinal and mesenteric lymph nodes, respectively).

There was no significant relationship between pre-challenge VN or calculated ELISA titres and any post-challenge viremia or virus excretion values.

## Discussion

Characterization of new PCV2 field isolates is important for many reasons, (i) understanding its infection dynamics, (ii) suitability for PCV2 vaccine efficacy evaluation and (iii) testing the suitability of relevant diagnostic methods. The objective of this small scale study was to characterize a recent isolate of the PCV2d-2 genotype, which is becoming dominant worldwide, by using quantifiable parameters, i.e. viraemia, virus shedding, and viral load in lymph nodes measured by qPCR. Different assays to monitor humoral immune responses upon challenge with the isolate were also tested.

Viral challenge evoked humoral antibody responses, which was demonstrated by all tested assays. The strongest correlation was found between the VN assay and the BioChek ELISA. Using the Circovirus IgM/IgG ELISA proved to be a good addition to follow the dynamics of infection besides measuring virus excretion and viremia. The observation that two pigs did not react positive in the INGEZIM IgM/IgG ELISA but were viremic and shed the virus could indicate (i) initial tolerance of the host towards PCV2, as suggested by Klausmann et al. [16], and/or (ii) the lower sensitivity/specificity of INGEZIM IgM/IgG ELISA compared to BioChek ELISA or VN assay for example (see Fig. 2) in this challenge model.

PCV2d genotype strains are reported to have earlier onset of viremia and enhanced virulence than PCV2b strains in experimentally infected pigs [14, 17]. This increased virulence is tentatively attributed to an extra lysine (K) residue in the capsid protein, making it 234 amino acids (aa) long, as compared to the 233 aa capsids of PCV2a and b strains [14].

Our own unpublished observation was in alignment with this finding (data not shown).

In the present trial, the PCV2 load in the lymph nodes (mediastinal and mesenteric) collected from the challenged animals was above 10^6^/500 ng of tissue, which meets this criteria of “PCV2 systemic disease” [18]. However, it is difficult to assign specific viral loads to a disease status as many factors might affect it, including genetics, co-infections, antibody status, and the time passed after infection.

Concerning the monitoring of infection dynamics, the results presented here were in agreement with that of Patterson et al. [19], who demonstrated peak viremia and shedding between 14 and 19 days post infection (dpi) in pigs experimentally infected with a PCV2b strain, while it was day 28 post farrowing in pigs naturally exposed to the same strain [20]. Regarding sample matrices, rectal swab sampling proved to be highly sensitive for detecting the virus early after infection in this challenge model (intranasal route of challenge).

Individual rectal swabs were used to measure virus excretion in this trial and this sample type was found more practical and less prone to cross-contamination then saliva collection on individual base. However, oral fluids are rather useful and informative samples, particularly on pen or herd level monitoring purposes [21].

## Conclusions

The results of this trial confirmed the potential application of the D3276/5/16HU PCV2d-2 isolate for experimental challenge purposes and demonstrated that rectal swabbing, followed by quantitative PCR analysis, is practical and an early indicator of the PCV2 status of the pigs. The results of all ELISA tests correlated with that of the VN assay. Measuring isotype specific humoral immune responses, combined with viremia data, can provide important information on the course of infection.

## Abbreviations

aa: Amino acids
DNA: Deoxyribonucleic acid
dpch: Days post challenge
dpi: Days post infection
ELISA: Enzyme-linked immunosorbent assay
MDA: Maternal-derived antibody
MOI: Multiplicity of infection
OD: Optical density
ORF: Open reading frame
PBS: Phosphate buffered saline
PBTN: PBS with 0.05% Tween 20 and 0.02% sodium azide
PCV2: Porcine circovirus type 2
PCVD: Porcine circovirus disease
PRRSV: Porcine reproductive and respiratory syndrome virus
qPCR: Quantitative polymerase chain reaction
VN: Virus neutralization
